# Diagnostic challenges of an atypical presentation of ear and mastoiditis tuberculosis in a-5-year-old child in a developing country: A case report and review of literature

**DOI:** 10.1016/j.idcr.2023.e01815

**Published:** 2023-06-07

**Authors:** Elrika Anastasia Wijaya, Sally Mahdiani, Lina Lasminingrum, Heda Melinda Nataprawira

**Affiliations:** a Department of Child Health, Universitas Padjadjaran/Hasan Sadikin General Hospital, Bandung, West Java, Indonesia; b Department of Otolaryngology, Universitas Padjadjaran/Hasan Sadikin General Hospital, Bandung, West Java, Indonesia

**Keywords:** Children, Mastoiditis tuberculosis, Diagnosis

## Abstract

We report a case of a 5-year-old girl who came to Otolaryngology Outpatient Clinic of our hospital with the chief complaint of recurrent ear discharge which had started to appear since 3 years ago and had recurred every month despite repeated antibiotics and antifungals ear drops treatments, given by general practitioners and otolaryngologists. Gram staining, acid-fast staining and *Mycobacterium tuberculosis* culture examination from the discharge had never been conducted. There was a history of inadequate weight increment since she was 2 years old. Her uncle who often met her died about 6 months ago due to TB disease. She was initially diagnosed with otitis media from the previous clinicians before finally referred to our hospital due to the perforated tympanic membrane. Otoscope examination showed perforated tympanic membrane. CT scan showed right osteomastoiditis with cholesteatoma with destruction of right mastoid antrum and right ear bone. We did not find pale-looking granulomatous tissue or multiple perforated tympanic membrane. The patient was diagnosed with chronic suppurative otitis media (CSOM) of the right ear with right mastoiditis and right posterior auricle fistulae. Afterwards, canal wall up, canaloplasty, mastoid obliteration, tympanoplasty, and rotational flap right ear were performed. Histopathology examination revealed the presence of caseous necrosis and datia langhans cells. Based on her chronic illness supported by the histopathological findings, anti-tuberculosis therapy was commenced. No more ear discharge complained and her body weight started to increase after oral anti tuberculosis treatment.

## Introduction

Ear tuberculosis is a rare form of extrapulmonary tuberculosis (TB). Its prevalence was estimated to be < 0.1 % [1]. Even in countries with high prevalence of TB, ear TB is rarely found in children. Typical presentation of it are usually unilateral, painless otorrhea, and multiple small perforations of tympanic membrane, sometimes accompanied with peripheral facial palsy. When presented with unspecific presentation, ear TB might get hard to diagnose since the signs and symptoms are similar with common bacterial ear infection and as the implication, anti-tuberculosis drug might be given later than it should be [2]. Thus, clinicians need to be cautious of the possibility of a child with ear discharge to contract ear TB, especially when the complaint accompanied by signs and symptoms of tuberculosis such as inadequate weight gain, night sweating, prolonged cough, or history of contact with adult who has tuberculosis treatment, especially in developing countries with high TB prevalence.

Aside from the possible complication of the ear TB itself such as hearing loss and central nervous system paresis, delay in diagnosis and treatment initiation of TB might cause the disease to spread to more organs and it can be harmful for the children. Up to date, there was still very few case reports regarding ear TB, especially in children.

Indonesia is one of the countries with highest prevalence of TB, contributing to about 14 % cases in the world and ranked 2nd in the world. Despite the trend of decreased new TB case findings during 2020–2021 (during COVID-19 pandemic), the number of new TB infection is still pretty high [3].

This case illustration might very well pictures a rare case of ear TB infection in a child in a country with a high prevalence of TB.

## Case presentation

A-5-year-old girl with 17 kg weight and 104 cm height came to Otolaryngology Outpatient Clinic with the chief complaint of yellow-greenish, foul smelling discharge from right ear. The complaint had lasted for about 3 years. There was also a nonpainful post aural swelling with discharge for the past 6 months. The parents also complained that the patient’s weight was decreased about 1 kg in the past 2 months. She had a history of inadequate weight increment. There was no night sweating, cough for more than 2 weeks, or low grade fever for more than 2 weeks. The patient had a history of tuberculosis contact with her father and uncle. The father underwent TB treatment at about 7 year ago but was declared to be healthy at the end of the 6 months treatment 2 years before the patient was born. The patient’s uncle died due to TB about 6 months ago and had already had TB treatment for about 2 months. Contact investigation for TB had not been done.

The patient had been brought to general practitioners and otolaryngologists since the complaint started. The complaint usually resolved for about 1 month after the patient had eardrops prescribed by the previous clinicians, but it usually happened again every 3–4 months since 3 years ago. On the last examination with the otolaryngologist about 6 months ago, the patient was referred to the district hospital due to the perforated tympanic membrane and from there, the patient was finally referred to our hospital. On the physical examination, we found post aural swelling about 2 × 3 cm, mobile with no pain. There was some greenish discharge came out from the right ear. Other physical examinations were within normal limits.

The blood examination and chest X-ray revealed no abnormality. On physical examination, mucopurulent ear discharge and hyperemic posterior auricle at right ear were found. There was no cranial nerve paresis. Audiometry examination revealed moderate conductive hearing loss at right ear (33.75 dB) and no hearing loss at left ear. Head CT scan with contrast was done and the expertise showed right osteomastoiditis with cholesteatoma which destructed right mastoid antrum and right auricle bone. The presence of right posterior auricle abscess was also found. The patient was initially diagnosed with chronic otitis media suppurative right auricle with right mastoiditis and right posterior auricle fistulae.

Afterwards, canal wall up mastoidectomy to remove mastoid air cell and mastoid obliteration continued with canaloplasty to widen ear canal, tympanoplasty to repair tympanic membrane, and rotational flap on the right auricle to close the defect were done. Intraoperatively, we found posterior auricle fistulae, dehiscence with cholesteatoma and granulous tissue at mastoid, subtotal tympanic membrane perforation and destruction of ossicles. Histopathology examination was done on the samples obtained from the ear canal. Upon examination, caseous necrosis and datia langhans cells were found. Due to the histopathological findings, the patient was referred to the pediatric pulmonology division. Mantoux test was done, resulting in 25 mm induration suggesting TB infection. GeneXpert test was done on the patient’s gastric lavage sample, and no M. tuberculosis was found. Acid fast staining examination for three times also revealed no M. tuberculosis bacteria in the patient’s gastric lavage samples.

The patient was treated using pediatric fixed dose combination tablets intensive phase (isoniazid, rifampicin, pyrazinamide) added with ethambutol for 2 months based on the standard protocol treatment for extrapulmonary tuberculosis. Afterwards, the therapy was continued with fixed dose combination maintenance phase (isoniazid and rifampicin). The whole treatment was given for 6 months duration. After the therapy was done for 1 month, the patient was evaluated and the body weight was increased 0.5 kg. There was no more discharge coming out from the patient’s right ear. The patient had no more complaint after the medication had been completed for 6 months. Upon the completion of the therapy, total weight gain was 3 kg and she was in a healthy condition.

## Discussion

Tuberculosis cases around the world was estimated to be about 10 million cases. In 2020, based on the WHO Global Tuberculosis Report, Indonesia ranked 3rd (8.6 %) among the countries with the largest contribution of the TB incident cases worldwide, below India (26 %) and China (8.5 %) [4]. Primary tuberculosis found in the ear canal is usually caused by secondary infection from lungs, larynx, pharynx, or nose [5].

Theoretically, ear TB can happen through hematogenous spread from other tuberculous foci, direct extension from eustachian tube through mucous aspiration, and also direct implantation from tympanic membrane perforation [6]. The typical presentations of ear TB, specifically mastoiditis TB are usually unilateral, painless otorrhea, and multiple small perforations of tympanic membrane, with pale granulomatous tissue and sometimes accompanied with peripheral facial palsy, persistent pronounced dilation of vessel in anterior part of tympanic membrane which might persist for a long time, and protrusion of posterior part of tympanic membrane. However, the clinical presentation might have changed over the years nowadays. Some of them are significant otalgia and serous otorrhea which might get purulent when there is secondary bacterial infection. Sensorineural, mixed, or conductive hearing loss ranging from mild to severe are also some possible clinical manifestations of ear TB. Middle and external ears are sites which are more often affected. Destruction of bones and fallopian canal happens in mastoiditis TB even more often than the finding of cholesteatoma [7]. In mastoiditis TB, tympanic membrane perforation often occurs in the area of granulation due to the coalescence of the granuloma. Granulomatous process might also cause ossicles destruction and as the disease progresses, the increased amount of granulomatous tissue might cause blockage in the ear canal and caused hearing loss of various spectrum. Central nervous system paresis might also happen due to the direct extension of the disease [1].

Peripheral facial paralysis, retroauricular fistulae, labyrinthitis, meningitis, tuberculous osteomyelitis, cerebral or cerebellar abscesses, or even cellulites are some of the complications of the mastoiditis TB [8], [9]. A case report of mastoiditis TB in a 2-year-old child in United Kingdom showed intracranial complication of atypical mastoiditis TB, which is rarely found and caused the patient to have sudden deterioration, altered consciousness, drowsiness and lethargy. The symptoms persisted despite intravenous antibiotics treatment and TB was diagnosed through MTB culture found from the pus drained during surgical drainage. When OAT was started, the symptoms started to resolve [10]. Ear TB is indeed often hard to diagnose due to its often atypical presentation.

Surgical treatment showed better results compared to depend solely on the anti-tuberculosis drugs. Surgical treatment was similar with the treatment of chronic suppurative otitis media, with or without cholesteatoma [5], [11].

In this case, the presentation of mastoiditis TB is not typical. As previously mentioned, in mastoiditis TB, some of the pathognomonic presentation are persistent pronounced dilation of vessel in anterior part of tympanic membrane which might persist for a long time, protrusion of posterior part of tympanic membrane, pale granulomatous tissue, and the most common presentation is multiple perforations of tympanic membrane which are lacking in this patient and complicate the initial diagnostic process [12].

Ear TB might not be life threatening, but it still needs to be diagnosed and treated as early as possible to persevere the patient’s quality of life due to its many possible complications when untreated for a long time. Thus, whenever we find ear discharge as a clinical presentation in children especially in TB endemic countries, GeneXpert Tuberculosis, gram staining, acid fast staining and M. tuberculosis culture should be considered to be examined from the ear discharge sample, so that early diagnosis and treatment in ear TB can be done. Upon diagnosis, especially when accompanied with central nervous system involvement, the presence of disseminated tuberculosis and immunocompromise underlying disease should be ruled out. Work up on tuberculosis should be done accordingly, such as mantoux test, chest X-ray, gastric lavage or sputum examination and lumbar puncture when needed. The patient’s HIV status needs to be obtained too [10].

Histopathology examination plays and important role in diagnosing ear TB. Granulation tissue biopsies might reveal typical findings of TB such as caseous necrosis and specific granulation with epitheloid and giant Langerhans cells, similar with the findings in our case. When these findings are found, anti tuberculosis treatment needs to be started immediately. When available, PCR genetic from the biopsy material can also be done. M. tuberculosis culture also needs to be done. Besides histopathology and PCR examination, imaging is also important to determine the degree of ear destruction before deciding the need to do surgical treatment in ear TB patient [5], [8].

Ototoxicity of ethambutol should be of a concern when giving anti-tuberculosis treatment in children with ear TB. Routine hearing examination should be done before, during and upon completion of the therapy [6].

**Fig. 1:**
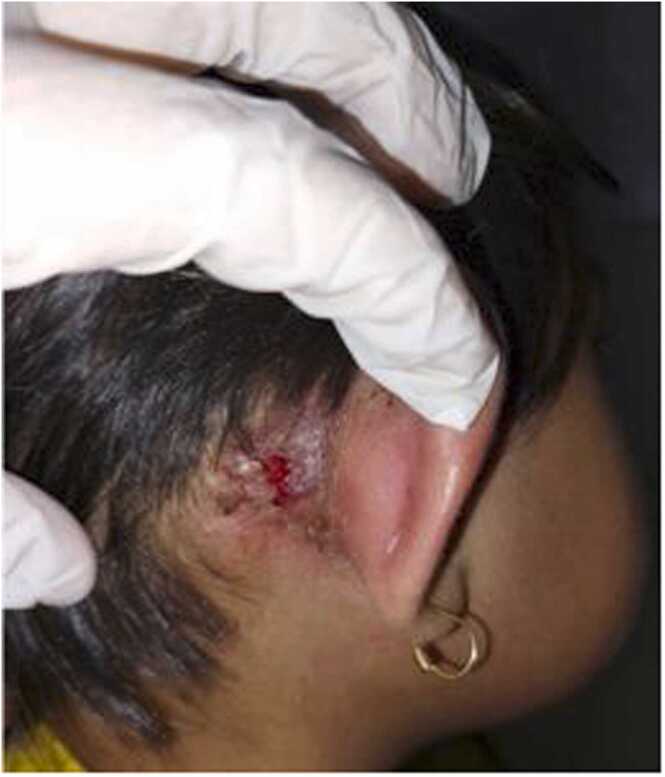
Right post auricular clinical presentation upon initial admission in Otolaryngology Outpatient Clinic.

**Fig. 2:**
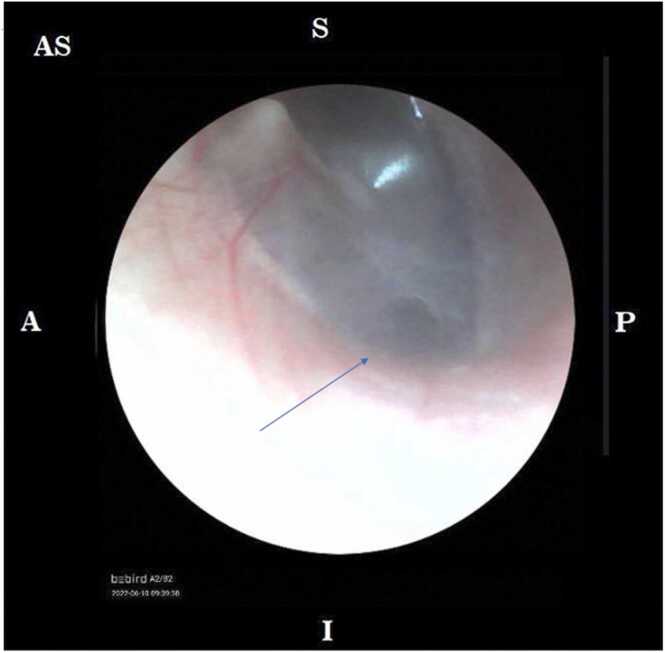
Tympanic membrane perforation seen through otoscope (blue arrow).

**Fig. 3:**
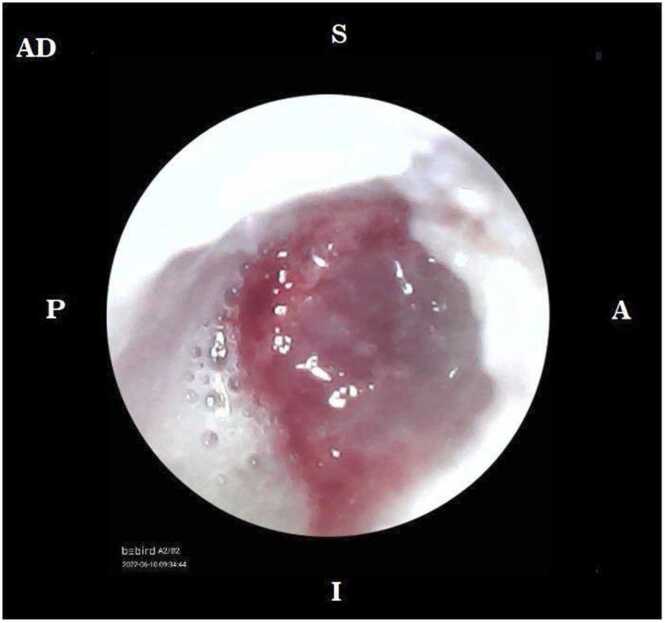
Granulomatous tissue inside the ear canal.

**Fig. 4:**
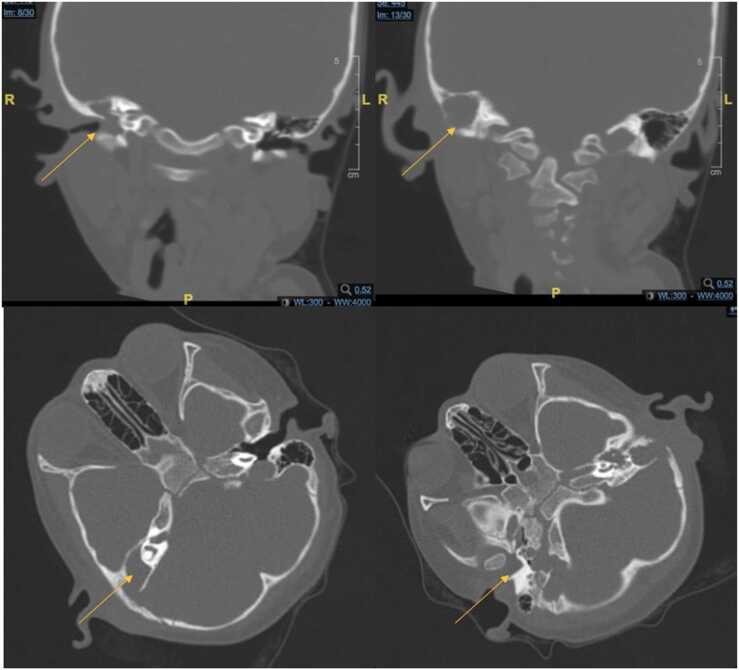
Head CT scan result revealed right osteomastoiditis with cholesteatome which destructed right mastoid antrum, right ear ossicle destruction, and also right posterior auricular abscess (yellow arrow).

**Fig. 5:**
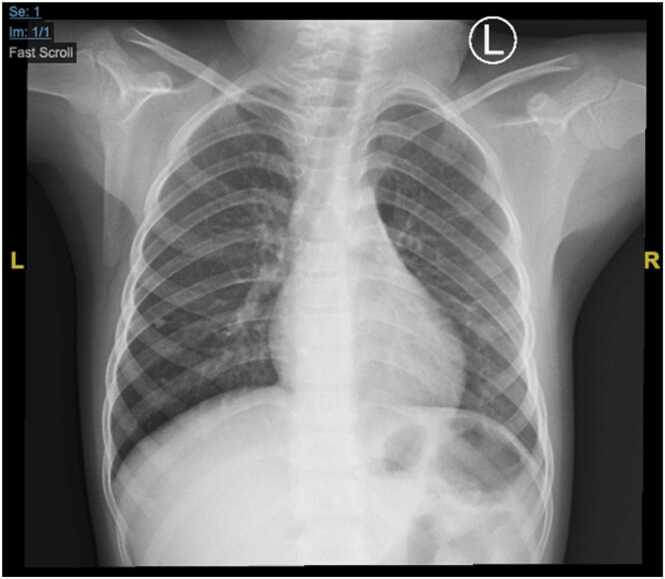
Chest X-Ray revealed normal appearance.

History of tuberculosis contact was clear in this patient. However, in our case, contact investigation had not been done yet. Contact investigation is very important in end TB strategy with the purpose of identifying and treating the source of TB transmission and preventive treatment should be given when needed [13]. However, it was still not routinely implemented due to various reasons, such as low awareness, illiteracy, and fear of stigma from society, and limited knowledge of tuberculosis on both caregivers and the patient [14]. Education regarding the importance of contact investigation for the society and local caregivers, especially in highly endemic countries need to be done to increase active case findings and treat the disease as early as possible through contact investigation. Fig. 1, Fig. 2, Fig. 3, Fig. 4, Fig. 5.

## Conclusions

Knowledge about ear TB is important for clinicians despite the rarity of the case. CT scan and histopathological examination are important modalities to diagnose ear TB. Adequate therapy can affect the child’s outcome, prevent the spread of the disease, preserve the hearing function, and increase the patient’s quality of life in the long term. The most importantly, whenever ear discharge is found as the clinical presentation especially when accompanied with classic clinical presentations of tuberculosis, we highly advise for GeneXpert Tuberculosis, gram staining, acid fast staining and M. tuberculosis culture to be done from the ear discharge, so that if it is caused by TB, the disease can be detected and treated as early as possible, especially when the case is found in highly endemic countries. Treatment should be done based on the TB treatment for extrapulmonary TB, using standard regiment for children consists of 2 months intensive phase with isoniazid, rifampicin, pyrazinamide, and ethambutol continued with maintenance phase with isoniazid and rifampicin for 4 months, with evaluation of the therapy efficacy during and after the treatment.

## Funding

The authors thank Universitas Padjadjaran for the financial help granted in publishing this manuscript.

## Ethics approval

This study adhered to the Declaration of Helsinki and written consent to publish this study had been obtained from the patient’s parents.

## Consent

Written informed consent was obtained from the patient's parents for publication of this case report and accompanying images. A copy of the written consent is available for review by the Editor-in-Chief of this journal on request

## CRediT authorship contribution statement

**Elrika Anastasia Wijaya (EAW):** drafted the study and created the content of the manuscript. **Sally Mahdiani (SM):** revised and added the content of the manuscript, prepared the imaging report, and managed the patient's operative procedure. **Lina Lasminingrum (LL):** did the literature research and revised the content of the manuscript. **Heda Melinda Nataprawira (HMN):** drafted the study, supervised the making of the manuscript and was involved in the patient’s management.

## Conflict of interests

The authors declare that the research was conducted in the absence of any commercial or financial relationships that could be construed as a potential conflict of interest.

## Data Availability

The data presented in this article are not readily available because they contain identifiable protected health information. Requests to access the datasets should be directed to Heda Melinda Nataprawira (heda.melinda@unpad.ac.id or heda_1155@yahoo.com).
